# Estimating Niche Width Using Stable Isotopes in the Face of Habitat Variability: A Modelling Case Study in the Marine Environment

**DOI:** 10.1371/journal.pone.0040539

**Published:** 2012-08-02

**Authors:** David O. Cummings, Camille Buhl, Raymond W. Lee, Stephen J. Simpson, Sebastian P. Holmes

**Affiliations:** 1 School of Biological Sciences, University of Sydney, Sydney, New South Wales, Australia; 2 School of Biological Sciences, Washington State University, Pullman, Washington, United States of America; 3 The School of Natural Sciences, The University of Western Sydney, Penrith, New South Wales, Australia; 4 Cardno Ecology Lab, Cardno NSW/ACT Pty Ltd, St Leonards, New South Wales, Australia; National Institute of Water & Atmospheric Research, New Zealand

## Abstract

Distributions of stable isotopes have been used to infer an organism's trophic niche width, the ‘isotopic niche’, and examine resource partitioning. Spatial variation in the isotopic composition of prey may however confound the interpretation of isotopic signatures especially when foragers exploit resources across numerous locations. In this study the isotopic compositions from marine assemblages are modelled to determine the role of variation in the signature of prey items and the effect of dietary breadth and foraging strategies on predator signatures. Outputs from the models reveal that isotopic niche widths can be greater for populations of dietary specialists rather than for generalists, which contravenes what is generally accepted in the literature. When a range of different mixing models are applied to determine if the conversion from δ to p-space can be used to improve model accuracy, predator signature variation is increased rather than model precision. Furthermore the mixing models applied failed to correctly identify dietary specialists and/or to accurately estimate diet contributions that may identify resource partitioning. The results presented illustrate the need to collect sufficiently large sample sizes, in excess of what is collected under most current studies, across the complete distribution of a species and its prey, before attempts to use stable isotopes to make inferences about niche width can be made.

## Introduction

**Table 1:** Depths (metres) and distance (kilometres) between the sites.

Wellhead	Goodwyn	Wanaea	Yodel	Cossack
Depth	136 m	84 m	137 m	82 m
**Distance between well heads**				
Wanaea	45 km			
Yodel	31 km	74 km		
Cossack	55 km	10 km	84 km	

Stable isotope analysis is often used by ecologists to identify trophic interactions [1]. This approach can be less problematic than others such as gut analysis, which may have logistical constraints and require regular and large sampling regimes [2], [3]. In the last decade, a number of authors have used stable isotopes to estimate trophic niche width [1], [3] and to examine resource partitioning [4], [5]. There has, however, been a growing realisation that interpreting patterns of stable isotope relies heavily on a comprehensive understanding of habitat use by predators, and the spatial patterns of isotopic variation among organisms at all trophic levels [6], [7], [8], [9], [10]. Post [3] has concluded, that without a suitable quantification of the isotopic composition of prey items, comparisons of consumers among and across habitats will be confounded by variations in prey signatures. The challenge for ecologists is to determine where isotopic variation exists and why.

An assumption of many studies aiming to estimate isotopic niche breadth, developed from the niche variation hypothesis proposed by Van Valen in 1965 [11], is that niche width correlates positively with diet breadth [12]. In this case, dietary specialists, i.e. those that utilise only a small number of food types at the population level, will have a narrow isotopic niche width, whereas dietary generalists, i.e. those that utilise a wide range of food resources at the population level, will have a broad isotopic niche width [11], [13], [14]. More recently this assumption has been challenged by studies which indicate that the converse can be true, i.e. the isotopic niche width of specialists can be broader than that for generalists, and that habitat use may complicate any conclusions that can be drawn from isotopic data [15], [16]. In addition, variation in isotopic signatures in δ-space (the dimensional space occupied by two or more isotopic signatures) may lead to incorrect estimates of the range of resources a population utilises. One suggestion to overcome this is to convert isotopic signatures from δ to p-space (relative proportions of prey items contributing to delta space signature) [17], [18]. The transformation to dietary proportions (p-space) is thought to resolve scaling discrepancies in δ-space, allowing direct comparison with a metric based measure of niche width [1], [19].

**Table 2:** Values of δ13C and δ15N (mean and standard deviation) for the prey species collected from each site.

		Global			^Goodwyn^			^Yodel^			^Echo^			<@emph type="sup">Wanaea<@/emph>		
^Prey^		^n^	^C13δ^	^N15δ^	^n^	^C13δ^	^N15δ^	^n^	^C13δ^	^N15δ^	^n^	^C13δ^	^N15δ^	^n^	^C13δ^	^N15δ^
^Pseudanthias rubrizonatus*^	^Reef Fish^	^195^	^−17.80 (0.63)^	^11.25 (0.88)^	^95^	^−17.99 (0.69)^	^11.0 (0.99)^	^37^	^−17.71 (0.49)^	^11.62 (0.89)^	^8^	^−17.84 (0.54)^	^11.54 (0.87)^	^55^	^−17.51 (0.50)^	^11.40 (0.46)^
^Rhynchocinetes balssi*^	^Shrimp^	^34^	^−16.29 (0.41)^	^11.34 (0.83)^	^10^	^−16.54 (0.20)^	^11.89 (0.27)^	^10^	^−16.48 (0.29)^	^11.85 (0.34)^	^4^	^−15.77 (0.58)^	^11.58 (0.22)^	^10^	^−16.04 (0.31)^	^10.17 (0.38)^
^Petrolisthes militaris*^	^Crab^	^36^	^−18.10 (1.39)^	^10.77 (0.81)^	^10^	^−16.19 (0.25)^	^11.37 (0.40)^	^9^	^−18.49 (0.57)^	^10.60 (0.33)^	^8^	^−18.65 (0.42)^	^11.27 (0.36)^	^8^	^−19.44 (1.06)^	^9.62 (0.69)^
^Pilumnus scabriusculus*^	^Crab*^	^31^	^−16.82 (0.64)^	^11.22 (0.87)^	^10^	^−17.15 (0.40)^	^11.27 (0.44)^	^9^	^−16.68 (0.35)^	^12.15 (0.42)^	^7^	^−16.13 (0.43)^	^10.14 (0.41)^	^5^	^−17.41 (0.78)^	^10.95 (0.76)^
*^Maja spinigera^*	^Spider Crab^	^16^	^−16.92 (0.61)^	^11.60 (1.52)^				^6^	^−17.06 (0.48)^	^11.58 (1.30)^	^8^	^−17.05 (0.63)^	^12.28 (1.58)^			
*^Portunus nipponensis^*	^Crab^	^7^	^−17.71 (1.54)^	^11.30 (0.51)^	^3^	^−19.33 (0.25)^	^10.86 (0.33)^	^4^	^−16.49 (0.34)^	^11.62 (0.33)^						
*^Pylopaguropsis pustulosa^*	^Hermit Crab^	^31^	^−17.87 (0.75)^	^9.78 (1.05)^				^9^	^−17.97 (0.30)^	^9.82 (0.32)^	^8^	^−18.41 (0.91)^	^11.2 (0.40)^	^14^	^−17.51 (0.48)^	^9.03 (0.87)^
*^Munida rogeri^*	^Squat Lobster^	^19^	^−17.29 (0.81)^	^11.19 (0.48)^	^10^	^−16.80 (0.52)^	^11.45 (0.45)^	^9^	^−17.83 (0.73)^	^10.90 (0.34)^						
*^Lysmata amboinensis^*	^Shrimp^	^18^	^−16.02 (0.76)^	^11.72 (0.74)^	^10^	^−15.62 (0.37)^	^12.03 (0.41)^	^5^	^−16.49 (1.04)^	^11.66 (0.95)^				^3^	^−16.55 (0.58)^	^10.80 (0.36)^
*^Lysmata sp.^*	^Shrimp^	^11^	^−17.05 (0.62)^	^11.26 (0.64)^	^8^	^−16.76 (0.21)^	^10.93 v(0.36)^	^3^	^−17.82 (0.74)^	^12.14 (0.17)^						
*^Alpheus gracilipes^*	^Shrimp^	^3^	^−16.53 (0.59)^	^11.48 (0.83)^	^3^	^−16.53 (0.59)^	^11.48 (0.83)^									
*^Paranthus sp.^*	^Anemone^	^29^	^−18.04 (1.71)^	^12.03 (0.66)^	^10^	^−16.20 (0.36)^	^11.87 (0.57)^	^10^	^−18.32 (0.34)^	^12.36 (0.84)^	^7^	^−19.75 (1.50)^	^11.74 (0.41)^			
*^Megabalanus tintinnabulum^*	^Barnacle^	^20^	^−18.57 (0.40)^	^10.02 (0.83)^	^8^	^−18.72 (0.18)^	^9.89 (0.36)^	^9^	^−18.52 (0.32)^	^9.89 (1.12)^	^3^	^−17.80 (0.40)^	^10.72 (0.31)^			
^Bait fish^	^Fish^	^8^	^−17.89 (0.23)^	^11.57 (0.24)^							^8^	^−17.90 (0.23)^	^11.60 (0.24)^			
*^Pseudanthias shemii^*	^Reef Fish^	^13^	^−18.25 (0.15)^	^11.96 v(0.53)^				^3^	^−18.29 (0.22)^	^12.29 (0.58)^	^8^	^−18.25 (0.15)^	^11.73 (0.50)^			
^Pseudanthias sp.^	^Reef Fish^	^5^	^−18.16 (0.63)^	^12.50 (0.30)^				^5^	^−18.16 (0.63)^	^12.50 (0.30)^						
^Gobiidae sp.^	^Fish^	^3^	^−18.13 (0.34)^	^11.39 (0.93)^	^3^	^−18.13 (0.34)^	^11.39 (0.93)^									
^Apogonidaesp.^	^Fish^	^3^	^−17.41 (0.71)^	^11.10 (0.52)^	^3^	^−17.41 (0.71)^	^11.10 (0.52)^									
^Turritellidae sp.^	^Gastropod^	^4^	^−15.68 (0.41)^	^12.66 (0.99)^				^4^	^−15.68 (0.41)^	^12.66 (0.99)^						
^Ranellidae sp.^	^Gastropod^	^5^	^−17.78 (0.09)^	^10.38 (0.33)^	^5^	^−17.78 (0.09)^	^10.38 (0.33)^									
^Ophiuridae sp.^	^Brittle Star^	^3^	^−16.91 (0.13)^	^10.40 (0.86)^	^3^	^−16.91 (0.13)^	^10.40 (0.86)^									

Global values were calculated from pooled site data. *Four common prey species.

**Figure 1:**
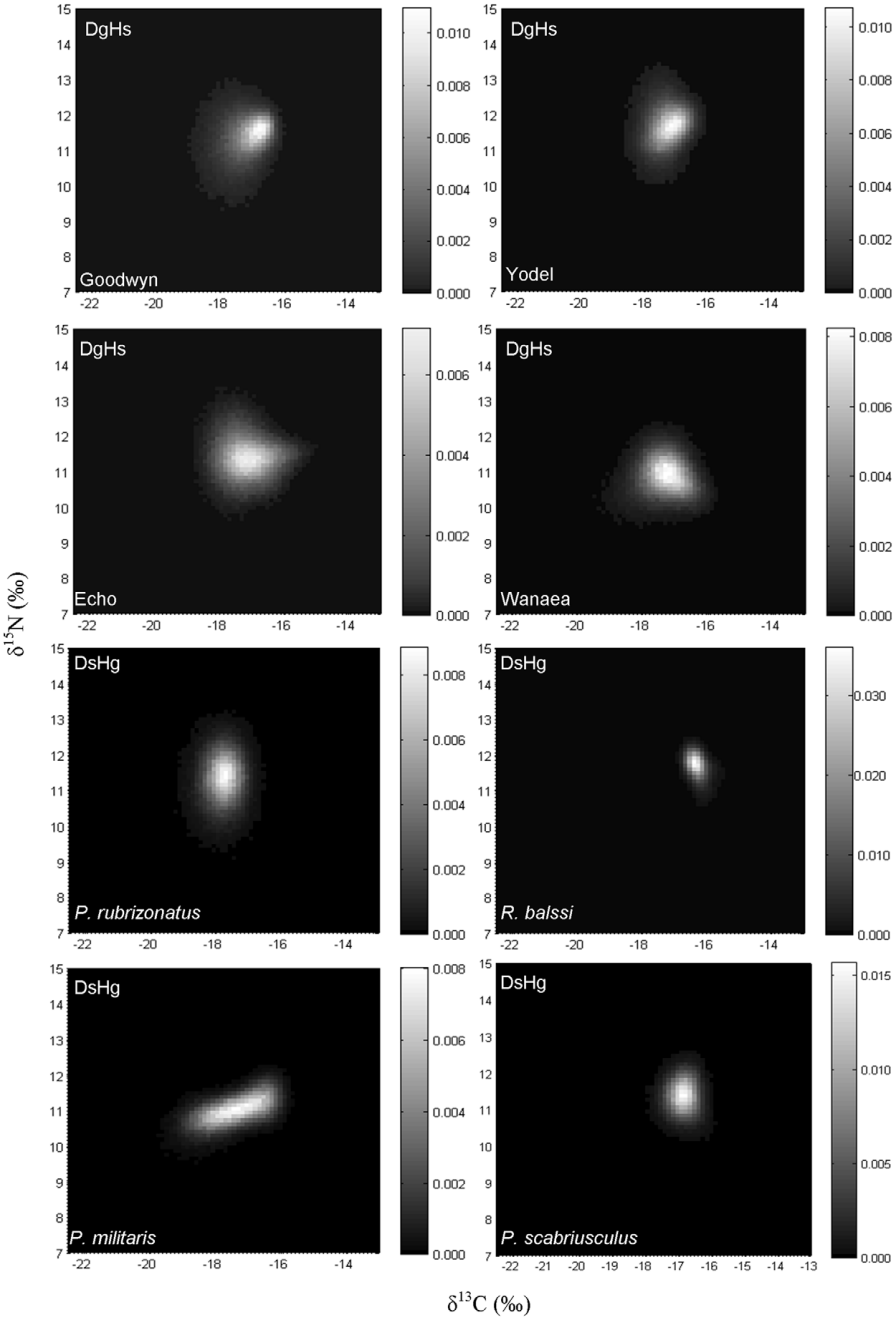
2D histograms showing the distribution of the results obtained for 100 000 of the isotopic signatures from the modelled Almaco Jack in δ-space for the common species (Part 1) for dietary generalists and habitat specialists (D_g_H_s_) and dietary specialists and habitat generalists (D_s_H_g_). Individual histogram greyscale bars indicate the relative frequency for each class.

**Figure 2:**
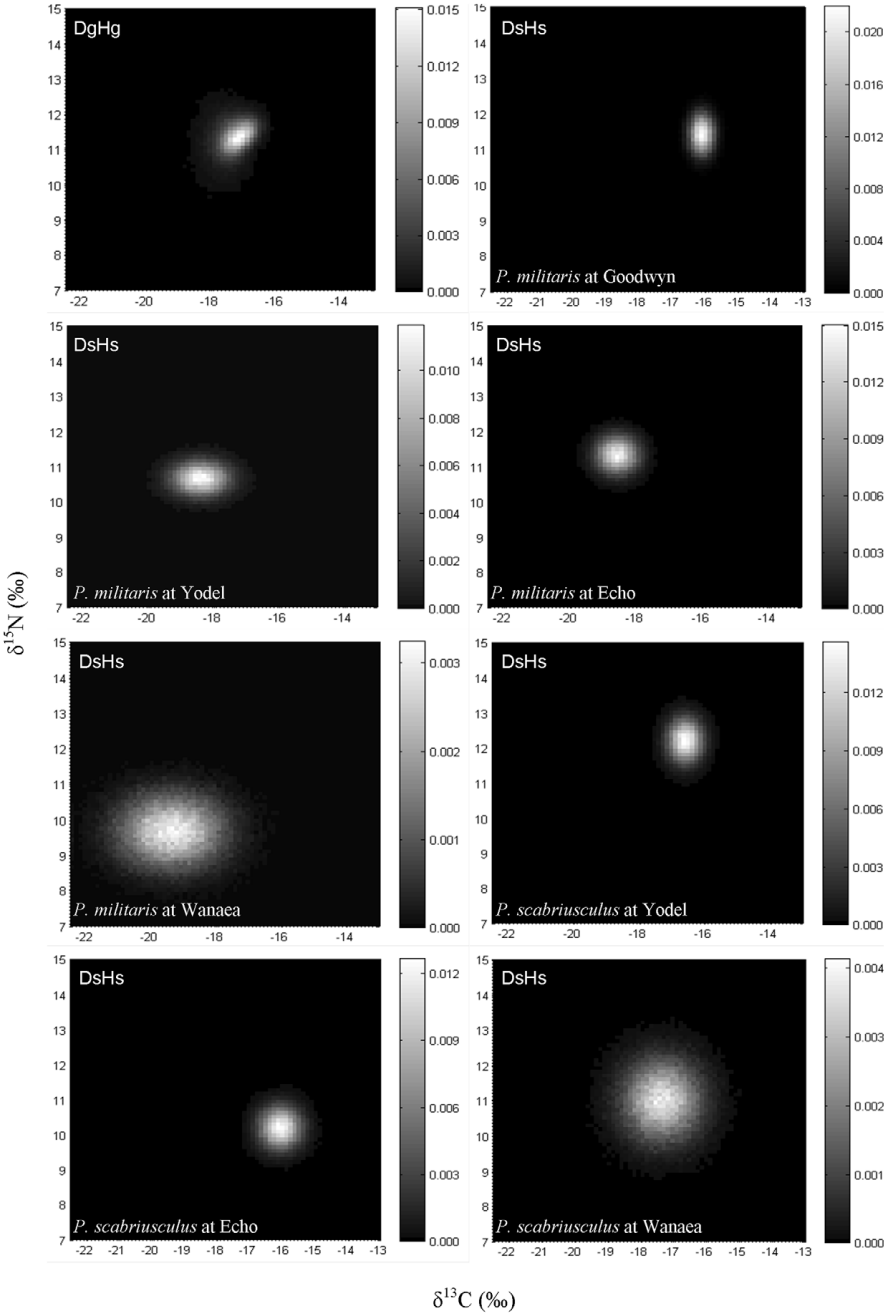
2D histograms showing the distribution of the results obtained for 100 000 of the isotopic signatures from the modelled Almaco Jack in δ-space for the common species (Part 1) for dietary generalists and habitat generalists (D_g_H_g_), habitat specialists that prey only on *Petrolisthes militaris* (D_s_H_s_) and habitat specialists that prey only on *Pilumnus scabriusculus* (D_s_H_s_). Individual histogram greyscale bars indicate the relative frequency for each class.

**Table 3:** Mean and variance of δ^13^C and δ^15^N for models of simulated Almaco Jack populations.

Model	Treatment	C^13^δ		N^15^δ	
Part 1		Mean	Variance	Mean	Variance
D_s_H_g_ (1)	*P. rubrizonatus*	−17.85	0.20	11.31	0.49
D_s_H_g_ (1)	*R. balssi*	−16.38	0.06	11.66	0.11
D_s_H_g_ (1)	*P. militaris*	−17.51	0.67	10.93	0.18
D_s_H_g_ (1)	*P. scabriusculus*	−16.90	0.14	11.32	0.21
**D_s_H_g_ (1)**	**Pooled**	**−17.16**	**0.58**	**11.30**	**0.31**
**D_g_H_g_ (2)**	**Generalist**	**−17.40**	**0.24**	**11.27**	**0.26**
D_s_H_s_ (3)	Goodwyn – *P rubrizonatus*	−17.96	0.45	11.00	0.97
D_s_H_s_ (3)	Goodwyn – *R. balssi*	−16.54	0.04	11.89	0.07
D_s_H_s_ (3)	Goodwyn – *P. militaris*	−16.19	0.07	11.36	0.17
D_s_H_s_ (3)	Goodwyn – *P. scabriusculus*	−17.17	0.14	11.27	0.20
D_s_H_s_ (3)	Yodel – *P. rubrizonatus*	−17.77	0.25	11.63	0.78
D_s_H_s_ (3)	Yodel – *P. balssi*	−16.47	0.09	11.85	0.12
D_s_H_s_ (3)	Yodel – *P. militaris*	−18.43	0.34	10.61	0.11
D_s_H_s_ (3)	Yodel – *P. scabriusculus*	−16.68	0.11	12.16	0.16
D_s_H_s_ (3)	Echo – *P. rubrizonatus*	−17.85	0.29	11.59	0.71
D_s_H_s_ (3)	Echo – *R. balssi*	−15.78	0.36	11.57	0.05
D_s_H_s_ (3)	Echo – *P. militaris*	−18.66	0.17	11.26	0.12
D_s_H_s_ (3)	Echo – *P. scabriusculus*	−16.12	0.16	10.11	0.18
D_s_H_s_ (3)	Wanaea *– R. rubrizonatus*	−17.50	0.25	11.39	0.23
D_s_H_s_ (3)	Wanaea – *R. balssi*	−16.04	0.09	10.13	0.14
D_s_H_s_ (3)	Wanaea – *P. militaris*	−19.47	1.20	9.58	0.49
D_s_H_s_ (3)	Wanaea – *P. scabriusculus*	−17.34	0.58	10.96	0.59
**D_s_H_s_ (3)**	**Pooled**	**−17.25**	**1.35**	**11.15**	**0.80**
D_g_H_s_ (4)	Goodwyn	−17.29	0.29	11.29	0.41
D_g_H_s_ (4)	Yodel	−17.36	0.23	11.55	0.32
D_g_H_s_ (4)	Echo	−17.17	0.45	11.33	0.32
D_g_H_s_ (4)	Wanaea	−17.34	0.35	10.80	0.28
**D_g_H_s_ (4)**	**Pooled**	**−17.29**	**0.34**	**11.24**	**0.41**

Part 2					
D_s_H_g_	*P. rubrizonatus*	−17.81	0.23	11.52	0.56
D_s_H_g_	*R. balssi*	−15.87	0.25	11.56	0.04
D_s_H_g_	*P. militaris*	−18.64	0.13	11.14	0.10
D_s_H_g_	*P. scabriusculus*	−16.24	0.15	10.37	0.12
**D_s_H_g_ (1)**	**Pooled**	**−17.14**	**1.47**	**11.15**	**0.43**
**D_g_H_g_**	**Generalist**	**−17.24**	**0.25**	**11.36**	**0.24**

Part 3					
**D_g_H_g_ (2)**	**Generalist**	**−17.33**	**0.14**	**11.34**	**0.18**
D_g_H_s_ (4)	Goodwyn	−17.23	0.31	11.31	0.34
D_g_H_s_ (4)	Yodel	−17.47	0.20	11.61	0.33
D_g_H_s_ (4)	Echo	−17.34	0.40	11.43	0.34
D_g_H_s_ (4)	Wanaea	−17.34	0.34	10.73	0.26
**D_g_H_s_ (4)**	**Pooled**	**−17.35**	**0.32**	**11.27**	**0.43**

Those models in bold elucidate mean values for each population based on diet (specialist vs. generalist) and habitat (Wellhead). Where a model consists of numerous variations (different specialisations) a ‘Pooled’ value is provided as an accumulative mean value for the model. Models for Part 1 used the four common prey species. Models for Part 2 used the common prey species and incorporating distance between sites. Models for Part 3 used the entire prey assemblage.

**Table 4:** Tukey's post hoc results comparing variances between models using the four common prey species (Part 1) for δ^13^C and δ^15^N.

Isotope	Comparison	DsHg	DgHg	DsHs
δ^13^C	DgHg	*		
δ^13^C	DsHs	NS	***	
δ^13^C	DgHs	NS	NS	*
δ^15^N	DgHg	***		
δ^15^N	DsHs	***	NS	
δ^15^N	DgHs	***	NS	NS

NS: no significant difference; asterisks indicate significant differences at *p<0.05, ** p<0.01 and *** p<0.001. A).

Flaherty and Ben-David [15] examined the effects of diet and habitat use on isotopic derived trophic niche width, in both δ-space and p-space, by modelling the isotopic composition of predators employing different feeding strategies. Their findings revealed that populations of dietary generalists display narrower isotopic niches than dietary specialists, suggesting that estimates from isotopic values of trophic niche may be confounded by habitat-derived differences (see also [12]). Our aim in this paper was to develop the models of Flaherty and Ben-David [15] by adding new degrees of ecological realism and statistical robustness by taking advantage of a rich new isotopic database, while also extending the models from a terrestrial to a marine context.

The isotopic data used for the modelling were derived from marine assemblages collected from artificial reefs (decommissioned oil drilling wellheads) on the North-West Shelf of Australia. These data offer several novel and significant features for such an investigation: a) the fauna sampled at each location was complete (i.e. isotopic signatures from the entire community were collected); b) the wellheads are replicated (the same) structures that differ in location and depth; and c) the wellheads had a range of species from a similar trophic level, representing a good system to investigate the effects of a predator that forages widely but without many additional differences between food patches. In addition, the wellheads were in deep-water locations (i.e. relatively unstudied) and the sample sizes for individual species were large (n = 4 – 195).

**Figure 3:**
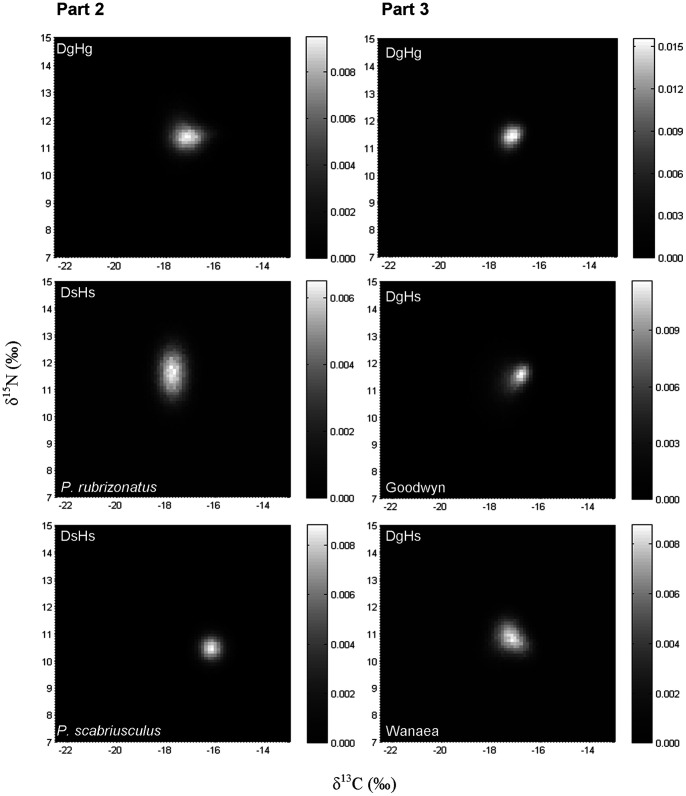
2D histograms showing the distribution of the results obtained for 100 000 of the isotopic signatures from the modelled Almaco Jack in δ-space incorporating distance between sites for the common prey species (Part 2), and the entire prey assemblages (Part 3). Models include those for dietary generalists and habitat generalists (D_g_H_g_), dietary specialists and habitat specialists (D_s_H_s_) (Part 2 only) and dietary generalists and habitat specialists (D_g_H_s_) (Part 3 only). Individual histogram greyscale bars indicate the relative frequency for each class.

**Table 5:** Tukey's post hoc results comparing variances between models using the common prey species and incorporating distance between sites (Part 2).

Isotope	Comparison	D_s_H_g_ *P. rubrizonatus*	D_s_H_g_ *R. balssi*	D_s_H_g_ *P. militaris*	D_s_H_g_ *P. scabriusculus*
δ^13^C	D_s_H_g_ – *R. balssi*	NS			
δ^13^C	D_s_H_g_ – *P. militaris*	***	***		
δ^13^C	D_s_H_g_ – *P. scabriusculus*	***	***	NS	
δ^13^C	D_g_H_g_	***	**	***	***
δ^15^N	D_s_H_g_ – *R. balssi*	***			
δ^15^N	D_s_H_g_ – *P. militaris*	***	NS		
δ^15^N	D_s_H_g_ – *P. scabriusculus*	***	NS	NS	
δ^15^N	D_g_H_g_	***	***	**	*

NS: no significant difference; asterisks indicate significant differences at *p<0.05, **p<0.01 and ***p<0.001.

The objective of this study is to use similar models as those applied by Flaherty and Ben-David [15] to determine effects of habitat variability in prey on the isotopic outcomes of the predator. We will apply both multi-source and Bayesian-based models to determine if trophic interactions, such as trophic niche widths and resource partitioning can be accurately estimated. To confidently address this hypothesis we will improve the modelling approach and test the resulting outcomes with more rigorous statistical analysis. In addition, the isotopic outcomes of our model predator will be tested under more ecologically realistic assumptions that represent conditions a predator is likely to face in the real environment. The basis of this approach adopted the four basic foraging models (as outlined in the methods) tested by Flaherty and Ben-David [15], using all combinations of dietary and habitat specialists or generalists. For this study, habitat generalists refers to those collected from a range of wellhead (artificial reef) locations. Isotopic differences were simulated by: 1) using four common prey species of known isotopic signatures at each location; 2) incorporating the effects of distance between sites on the signatures of the predator feeding on the common prey species at each habitat/location; and 3) using the entire assemblage of prey sampled within a similar trophic level at each site.

**Table 6:** Tukey's post hoc results comparing variances between models using the entire prey assemblage (Part 3).

Isotope	Comparison	D_g_H_s_ Goodwyn	D_g_H_s_ Yodel	D_g_H_s_ Echo	D_g_H_s_ Wanaea
δ^13^C	D_g_H_s_ Yodel	***			
δ^13^C	D_g_H_s_ Echo	***	***		
δ^13^C	D_g_H_s_ Wanaea	NS	***	***	
δ^13^C	D_g_H_g_	***	NS	***	***
δ^15^N	D_g_H_s_ Yodel	NS			
δ^15^N	D_g_H_s_ Echo	**	NS		
δ^15^N	D_g_H_s_ Wanaea	***	**	NS	
δ^15^N	D_g_H_g_	***	***	***	NS

NS: no significant difference; asterisks indicate significant differences at *p<0.05, **p<0.01 and ***p<0.001.

## Methods

Animals were collected in 2008 from the North West Shelf of Australia approximately 100km offshore from Dampier, Western Australia, from isolated wellhead structures (see Table 1). The wellheads were remotely severed and brought onboard a construction vessel as part of the decommissioning works, allowing organisms to be collected directly by hand from the structures (see [20] for full details). The wellheads had been in place for 12 – 16 years, such that they were colonised by extensive communities of deep reef species. δ^13^C and δ^15^N isotopes from muscle tissue were collected as a part of a trophic study of the wellhead communities. Where potential for carbonate tissue existed i.e. decapod exoskeleton, ground tissue samples were treated with 2N phosphoric acid. Isotope signatures of freeze dried tissue were measured from 0.5 mg material at Washington State University using an Isoprime isotope ratio mass spectrometer (IRMS) (for detailed methods see [21]. The data used included signatures from a range of fishes, crustaceans, molluscs, anthozoans, asteroids and ophiuroids, from 4 of the 5 wellhead sites (Yodel, Goodwyn, Echo, and Wanaea) as these were the most comprehensively sampled sites.

**Table 7:** Comparison of source proportion estimates for each of the common prey species and Shannon-Wiener information measure (H) means and variances in p-space, between mixing models (SISUS, IsoSource and MixSIR) for Part 1 and 2.

Models		Prey sources				p-space	
Part 1	Mixing model	*Pseudanthias* *rubrizonatus*	*Rhynchocinetes* *balssi*	*Petrolisthes* *militaris*	*Pilumnus* *scabriusculus*	H_mean_	H_varianc_
D_s_H_g_	SISUS	0.30 (0.06)	0.44 (0.08)	0.11 (0.02)	0.15 (0.01)	−0.98	0.10
n = 8	IsoSource	0.35 (0.11)	0.33 (0.08)	0.12 (0.02)	0.19 (0.01)	−0.97	0.16
	MixSIR	0.29 (0.15)	0.09 (0.02)	0.26 (0.05)	0.36 (0.09)	−0.79	0.09
	SIAR	0.21 (0.01)	0.10 (0.01)	0.51 (0.03)	0.16 (0.01)	−1.08	0.04
	**Mean**	**0.29 (0.08)**	**0.24 (0.19)**	**0.25 (0.03)**	**0.22 (0.01)**	**−0.96**	**0.01**
D_g_H_g_	SISUS	0.36 (0.05)	0.15 (0.06)	0.28 (0.09)	0.20 (0.09)	−1.11	0.07
n = 7	IsoSource	0.31 (0.06)	0.13 (0.01)	0.24 (0.08)	0.19 (0.01)	−1.14	0.06
	MixSIR	0.11 (0.03)	0.22 (0.03)	0.45 (0.06)	0.22 (0.05)	−0.95	0.01
	SIAR	0.23 (0.04)	0.10 (0.01)	0.45 (0.02)	0.22 (0.01)	−1.14	0.01
	**Mean**	**0.25 (0.05)**	**0.15 (0.03)**	**0.36 (0.06)**	**0.21 (0.04)**	**−1.09**	**0.04**
D_s_H_s_	SISUS	0.09 (0.01)	0.49 (0.09)	0.10 (<0.01)	0.33 (0.10)	−0.88	0.11
n = 8	IsoSource	0.10 (<0.01)	0.47 (0.08)	0.09 (<0.01)	0.33 (0.08)	−0.95	0.07
	MixSIR	0.19 (0.09)	0.17 (0.02)	0.55 (0.05)	0.08 (0.01)	−0.88	0.06
	SIAR	0.06 (<0.01)	0.02 (<0.01)	0.90 (<0.01)	0.03 (<0.01)	−0.99	0.02
	**Mean**	**0.11 (0.03)**	**0.29 (0.05)**	**0.41 (0.01)**	**0.19 (0.05)**	**−0.93**	**0.07**
D_g_H_s_	SISUS	0.23 (0.04)	0.28 (0.04)	0.31 (0.04)	0.18 (0.01)	−1.09	0.04
n = 20	IsoSource	0.25 (0.03)	0.26 (0.04)	0.30 (0.04)	0.20 (0.01)	−1.15	0.01
	MixSIR	0.09 (0.01)	0.05 (<0.01)	0.79 (0.01)	0.07 (<0.01)	−0.66	0.02
	SIAR	0.21 (0.02)	0.12 (0.01)	0.60 (0.01)	0.07 (<0.01)	−0.40	0.02
	**Mean**	**0.20 (0.03)**	**0.18 (0.02)**	**0.50 (0.02)**	**0.13 (<0.01)**	**−0.83**	**0.02**

Part 2							
D_s_H_g_	SISUS	0.27 (0.02)	0.02 (<0.01)	0.69 (0.03)	0.03 (<0.01)	−0.72	0.09
n = 3	IsoSource	0.23 (0.02)	0.02 (<0.01)	0.71 (0.03)	0.04 (<0.01)	−0.74	0.09
	MixSIR	0.49 (0.18)	0.10 (0.01)	0.13 (0.02)	0.28 (0.09)	−0.84	0.01
	SIAR	0.26 (0.06)	0.16 (<0.01)	0.42 (0.07)	0.08 (<0.01)	−1.00	0.06
	**Mean**	**0.31 (0.07)**	**0.06 (<0.01)**	**0.49 (0.04)**	**0.11 (0.02)**	**−0.83**	**0.06**
D_g_H_g_	SISUS	0.46 (0.03)	0.12 (0.01)	0.25 (0.03)	0.17 (0.02)	−1.11	0.04
n = 9	IsoSource	0.45 (0.03)	0.12 (0.01)	0.26 (0.03)	0.17 (0.01)	−1.13	0.03
	MixSIR	0.36 (0.11)	0.06 (<0.01)	0.53 (0.08)	0.06 (0.01)	−1.13	0.03
	SIAR	0.28 (0.02)	0.13 (0.01)	0.41 (0.04)	0.12 (0.02)	−1.06	0.03
	**Mean**	**0.39 (0.05)**	**0.11 (<0.01)**	**0.36 (0.05)**	**0.13 (0.02)**	**−1.11**	**0.03**

Includes mean proportion estimates, and Shannon-Wiener means and variances for all three models, elucidated in bold.

**Table 8:** Comparison of variances in δ-space (for both δ^13^C and δ^15^N) with p-space (Shannon-Wiener information measure) for models using the common prey species (Part 1) and models using the common prey species and incorporated distance between sites (Part 2).

Part	Model	δ^13^C	δ^15^N	P
		D_s_H_g_ (*P. scabriusculus* only)		
Part 1	D_g_H_g_	1.7	1.2	2.4
Part 1	D_s_H_s_ (*P. scabriusculus* and Yodel only)	1.3	1.3	1.5
Part 1	D_g_H_s_ (Yodel only)	1.7	1.2	6.0
		D_g_H_g_		
Part 1	D_s_H_s_ (*P. scabriusculus* and Yodel only)	2.2	1.6	1.6
Part 1	D_g_H_s_ (*P. scabriusculus* only)	1.0	1.2	2.5
		D_s_H_s_ (*P. scabriusculus* and Yodel only)		
Part 1	D_g_H_s_ (Yodel only)	2.1	2.0	4.0
		D_s_H_g_ (*P. rubrizonatus* only)		
Part 2	D_g_H_g_	1.1	2.3	2.0

Using individual isotopic signatures, prey species of a similar trophic level (i.e. their δ^15^N signatures did not differ by more than 4‰) and common to all four of the selected sites (see Table 2) were identified. Generally, isotopic fractionation between trophic levels is assumed to be 3 – 4‰ [22].

### Foraging models

Models were created using the MATLAB software package. The large pelagic fish Almaco Jack (*Seriola rivoliana*) was chosen as a model predator in the simulations. Almaco Jack are known to feed opportunistically on a wide range of prey [23] including both fish and invertebrates [24], [25], and foraging across distances of up to 50 km [26] with the capacity to migrate hundreds of kilometres [27]. All parts of this study used the basis of the same modelling approach as Flaherty and Ben-David (2010), but to enhance reliability, modelling was based on 100 000 replicates per model rather than the 250 (see Text S1 for a detailed description of the model).

In the first part of this study to mimic the original model [15], four focal species; the fish *P. rubrizonatus* and decapods *R. balssi*, *P. militaris* and *P. scabriusculus* (see also [28]) common to all sites were designated as prey (Table 2), for four different predator models, as follows:

1. D_s_H_s_ – the predator is a dietary and habitat specialist (preys on specific items but has site fidelity) (Model 1);
2. D_s_H_g_ – the predator is a dietary specialist and habitat generalist (preys on specific items and forages between sites) (Model 2);
3. D_g_H_g_ – the predator is a dietary and habitat generalist (preys on everything and forages between sites) (Model 3);
4. D_g_H_s_ – the predator is a dietary generalist and habitat specialist (preys on everything but has site fidelity) (Model 4).

In part two of the study, the effect of distance between foraging sites on isotopic signatures of the Almaco Jack predator feeding on the four focal species was modelled. The aim was to model isotopic outcomes under conditions that are more likely to reflect a marine predator that is highly mobile and forages across large spatial scales. This was achieved by dictating the relative contribution of each habitat to reflect the effect of distance between foraging sites on habitat generalists (D_s_H_g_ and D_g_H_g_; see Text S1 for a detailed description of the model).

In the third part, to further increase ecological realism, entire prey assemblages at each site were used to reflect site composition (see Table 2). Hence, for this part, only dietary generalists (D_g_H_g_ and D_g_H_s_) were simulated. Unless otherwise denoted niche width is equal to the variance produced by the models.

### Data analysis

To determine if the common invertebrates varied in isotopic signatures among sites, a Multiple Analysis of Variance (MANOVA) was performed. All statistical tests were performed using the SPSS statistical package. The dependent variables δ^13^C and δ^15^N were compared among the fixed factors site and prey species. Variances were compared separately for both δ^13^C and δ^15^N to determine the effects of habitat/location variability on isotopic composition in δ-space. An O'Brien's transformation 29] was applied to convert the variance data into a format suitable for Analysis of Variance (ANOVA), as follows:



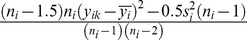



Where *n_i_* is the number of observations of group *i*, *y_ik_* is the *k* th observation of group *i*, 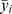 is the mean of the observations of group *i*, and 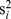 is the variance of the observations of group *i*.

In order to avoid Type II error (i.e. falsely accept the null hypotheses) rarefaction curves were generated to determine the optimal sampling size of modelled variances [30]. For the modelled data it was found that optimal sample size ranged between 100 and 950 observations, hence a median of 475 observations was randomly selected from the 100 000 modelled observations for analysis of their means and variances.

To compare the four models, all combinations relevant to that model were pooled. For example, for the model D_s_H_g_ this includes each of the prey species, which equates to four combinations). Analysis of Variance (ANOVA) was employed to test for differences between models, followed by Tukey's post hoc comparisons to identify where significant differences between models existed. However, the pooling of scenarios for models may confound some comparisons (i.e. where the effects of scenarios are opposite within each model, such differences due to pooling will not be apparent). Therefore, additional analysis using a one-way ANOVA with Tukey's post hoc comparisons between all possible scenarios of each model was performed. This same procedure was followed for all three parts of the study.

Mixing models were applied to the data to determine if converting δ-space to p-space (proportion space) as proposed by Newsome et al [19], could reduce variance to more accurately estimate trophic niche width, and to identify resource partitioning. To model the effects of prey variability across habitats that a “naive researcher” may encounter, 50 Almaco Jack were randomly sampled from the simulated populations. A sample size of 50 (predators) was deemed appropriate following initial runs which determined that a sample size of >15, as used by Flaherty and Ben-David [15], was required because the mixing space derived from the four reef species in this study was smaller.

Following the procedures of Flaherty and Ben-David [15], we constructed mixing spaces using the four focal prey species and selected Almaco Jack that fell only within this mixing space from simulated populations in both Part 1 and 2 for conversion to p-space. For models involving habitat generalists (D_g_H_g_ and D_s_H_g_), global means of the four common prey species (sources) were used to distinguish the mixing space. However global means for habitat specialists were deemed inappropriate as they fell outside the mixing space, therefore the appropriate site means were used (Table 2).

In addition to the multi-source mixing model SISUS [31] applied by Flaherty and Ben-David [15], we also used the IsoSource [32], SIAR [33] and MixSIR [34] models to convert variances to p-space and estimate proportions of prey species contributions. Unless otherwise denoted, the model default settings were used and no trophic enrichment factors (TEF's) were defined other than program defaults, where appropriate. For the SISUS (Bayesian based) model [14], 10 000 samples were selected to be retained for analysis within the model, which generated mean proportions and variances for each of the mixtures (fractions of prey contributing to predator signature). For the IsoSource model (multiple source dual isotope mixing-model) [32], an increment of 1% and tolerance of 0.05 were selected for each possible mixture to generate mean proportions and variances. For the MixSIR (Bayesian based) model [34] 1 000 000 iterations were run and a posterior density ratio of <0.01 was ensured. For the SIAR (Bayesian based) model [33], 1 000 000 iterations with a burnin of 400 000 iterations (“very long” default setting in the package) were run, standard trophic enrichment factors (TEF's) of 3.54‰ (standard devation (SD) of 0.74) for δ^15^N and and 1.63‰ (SD = 0.63) for δ^13^C for trophic level were used, no elemental concentration corrections and/or priors were defined. Mean proportions and variances were calculated by randomly selecting a number, equal to the sample sizes of the mixtures for any one scenario. Mixture sample size was determined from the number of predator signatures that fell within the two dimensional mixing space (defined by the delta values of the prey).

For models containing dietary and habitat specialists, *Pilumnus scabriusculus* and the Yodel site were randomly selected for part 1 (for D_s_H_g_ and D_s_H_s_), and *Pseudanthias rubrizonatus* in part 2 (D_s_H_g_). To determine the combined variances amongst proportions of each prey source in p-space, the Shannon-Wiener information measure (H) was used to estimate variances (niche width) [35]. These estimates were then compared with one way analysis of variance (ANOVA) followed by a Tukey's post hoc comparisons.

## Results

### Niche width estimates in δ-space

#### Part 1

The isotopic signatures of the common prey species varied among the sites (MANOVA, p<0.05; Table 2). Mean differences among sites were 0.5 ‰ (δ^13^C) and 0.6 ‰ (δ^15^N) for *Pseudanthias rubrizonatus*, 0.7 ‰ (δ^13^C) and 1.7 ‰ (δ^15^N) for *Rhynchocinetes balssi*, 3.2 ‰ (δ^13^C) and 1.8 ‰ (δ^15^N) for *Petrolisthes militaris*, and 1.3 ‰ (δ^13^C) and 2.1 ‰ (δ^15^N) for *Pilumnus scabriusculus*. Simulated models of Almaco Jack isotopic compositions from feeding on the common prey species (Part 1) found that their position within δ-space was variable (Fig 1, 2). In the majority of cases, higher variances indicated that dietary specialists (D_s_H_g_ and D_s_H_s_) occupied greater bivariate space than dietary generalists (D_g_H_s_ and D_g_H_g_). Pooled (i.e. the mean sum of all possible scenarios/combinations within each model) results for each model show that the isotopic niche can be greater for dietary specialists (D_s_H_g_ and D_s_H_s_) with variances of 1.7 to 5.6 and 2 – 3 times greater for δ^13^C and δ^15^N, respectively, than dietary generalists (D_g_H_s_ and D_g_H_g_) (Table 3). Comparison of O'Brien's variances among models with all possible scenarios pooled found significant differences for both δ^13^C (ANOVA, F_3, 11875_ = 8.27, *p*<0.001) and δ^15^N (ANOVA, F_3, 11875_ = 74.11, *p*<0.001). Post hoc comparisons revealed that for δ^13^C D_s_H_g_ populations had significantly greater variances than D_g_H_g_, while D_s_H_s_ variances were significantly greater than those of D_g_H_s_; however other comparisons e.g. D_s_H_s_ and D_g_H_s_, were not significantly different (Table 4). For δ^15^N, significant differences were only found for comparisons of D_s_H_g_ with all other models (Table 4).

A closer inspection of the modelled data for Part 1 revealed that the niche width displayed by the predator varied both among and within models (Fig 1, 2) (for additional plots see Figure S1). Further comparison among the modelled outcomes found that isotopic niche width varied between both sites and prey species for the simulated populations of Almaco Jack (Table 3). The data show that differences in isotopic variances of the predator are prey species specific. For D_s_H_g_, δ^13^C variances ranged from being 2.8 times greater to 4 times less than those of D_g_H_g_, while for δ^15^N, D_s_H_g_ variances ranged from 1.9 times greater to 2.4 times less than D_g_H_g_. In a similar manner the data reveal that for all models, differences in variances are prey source and/or habitat specific (see Table 3).

#### Part 2

The isotopic composition of habitat generalists was found to further vary when the distance that the predator travels between foraging sites was added to the model (See D_g_H_g_ Fig 3) (for additional plots see Figure S2). In δ-space the differences in variances of dietary specialists (D_s_H_g_) was variable between prey sources, ranging from being the same to 1.9 times greater than dietary generalists (D_g_H_g_) for δ^13^C, and 6 times less to 2.3 times greater than dietary generalists (D_g_H_g_) for δ^15^N (Table 3). Comparison of variances between models with scenarios pooled was significant for δ^13^C (ANOVA, F_1, 2375_ = 106.636, *p*<0.001) and δ^15^N (ANOVA, F_1, 2375_ = 6.083, *p*<0.05), while comparisons among all scenarios within each of the two models (1 and 4) were significant for both δ^13^C (ANOVA, F_4, 2375_ = 60.719, *p*<0.001) and δ^15^N (ANOVA, F_4, 2375_ = 208.679, *p*<0.001). All scenarios of dietary specialists (D_s_H_g_) were found to be different to dietary generalists (D_g_H_g_) for both δ^13^C and δ^15^N, while some comparisons between the different dietary specialists (D_s_H_g_) were also different (see Table 5).

#### Part 3

Differences in δ-space were also variable when comparing models of dietary generalists (D_g_H_g_ and D_g_H_s_) utilising the entire prey assemblages at each site (Fig 3; for additional plots see Figure S3). Variances of habitat specialists (D_g_H_s_) ranged from being 1.4 to 2.9 times greater than habitat generalists (D_g_H_g_) for δ^13^C, and 1.4 to 2.2 times greater than habitat generalists (D_g_H_g_) for δ^15^N (Table 3). Comparisons of pooled variances (i.e. those derived from the isotopic signatures) of simulated populations feeding on the entire prey assemblage were significant for both δ^13^C and δ^15^N isotopes (δ^13^C: ANOVA, F_1, 2375_ = 106.636, *p*<0.001; δ^15^N ANOVA, F_1, 2375_ = 66.189, *p*<0.001). Differences were also found for δ^13^C and δ^15^N variances among all scenarios within each of the two models compared (D_g_H_g_ and D_g_H_s_) (δ^13^C: ANOVA, F_4, 2375_ = 73.911, *p*<0.001; δ^15^N: ANOVA, F_4, 2375_ = 25.942, *p*<0.001). All comparisons of individual scenarios within D_g_H_g_ were different to D_g_H_s_ (with the exception of habitat specialists at Yodel for δ^13^C and Wanaea for δ^15^N), while only some comparisons between the different habitat specialists were different (see Table 6).

### Niche width estimates in p-space and prey source proportions

In Part 1, variances indicate that isotopic niche width in p-space was greater for the dietary specialists (D_s_H_g_ and D_s_H_s_), than the dietary generalists (D_g_H_g_ and D_g_H_s_) (Table 7), however only differences using the MIXSIR and SIAR models were found to be significantly different (SISUS: F_3, 43_ = 1.588, *p* = 0.208; IsoSource: F_3, 43_ = 2.082, *p* = 0.118; MIXSIR: F_3, 43_ = 5.013, *p*<0.05; SIAR: F_3, 43_ = 68.153, *p*<0.001). Post hoc comparisons for the MIXSIR model indicated that only dietary and habitat generalists (D_g_H_g_) were different from dietary generalist, habitat specialists (D_g_H_s_). In comparison, *post-hoc* analysis for the SIAR model revealed that dietary generalists and habitat specialists (D_g_H_s_) were different to all other categories, which were not different from each other (Table 8).

In Part 2 (where foraging distance was included in the models) the dietary specialist (D_s_H_g_) was found to have narrower isotopic niche than the dietary generalist (D_g_H_g_). Three of the four models found these differences to be significant, SISUS (F_1, 12_ = 6.220, *p*<0.05), IsoSource (F_1, 12_ = 6.794, *p*<0.05) and MIXSIR (F_1, 12_ = 6.794, *p*<0.05). These results should be interpreted with care as the sample size was small (Table 7). Results from both Parts 1 and 2 show that model variances decreased on conversion from δ-space. However, the differences in variances between models remained similar or increased (Table 8).

## Discussion

The results confirm that isotopic variability amongst habitats can confound estimates of isotopic niche in both δ-space and p-space. The modelling of isotopic compositions of simulated populations of Almaco Jack foraging between artificial reefs conforms with the terrestrial modelling by Flaherty and Ben-David [15]. In the present study, improved modelling techniques and more ecologically realistic conditions were applied to test the effects of isotopic variability between habitats on trophic niche width. In addition, data were converted from δ-space to p-space, as suggested by Newsome et al. [19] using a range of different mixing models to reduce scaling discrepancies. The modelling suggests that the isotopic variability of prey may confound any predictions of trophic niche, irrespective of an organism's trophic strategy (specialist vs. generalist) and/or the source of isotopic variation (spatial vs. compositional differences). In addition, the use of mixing models to convert δ-space variance to p–space variance offers little or no assistance. Interestingly, and in contrast to what is commonly accepted, although estimated isotopic niche breadth is a function of the variance of prey items (in this study global values of common prey species varied by 1.9‰ for δ^13^C and 0.5‰ for δ^15^N) and the spatial dispersion of that variance, dietary specialists appear to have a broader isotopic range than dietary generalists.

Analysis of the data revealed that prey variability in stable isotope signatures among habitats must be accounted for if we are to make realistic predictions about niche width. These results confirm that the natural variability that occurs across spatial scales of the study area will influence isotopic signatures, especially those of δ^13^C [16], [31], confounding comparisons of isotopic variances between many populations [36]. Natural variations in isotopic signatures will be evident amongst most basal resource pools. This is especially evident in the marine environment. For example phytoplankton are known to show trends of δ^13^C enrichment with decreasing latitude towards the equator [37], indicating fluctuations in the physiochemical environment may lead to variability. What remains clear, is that to interpret the variance amongst isotopic signatures of predators, isotopic variability of prey needs careful consideration [16], [38] and for many studies, adequate sampling across relevant spatial and temporal scales needs to be a prerequisite [39]. Despite this, a number of studies have attempted to estimate isotopic niche width as a measure of trophic niche [31], [40], [41], [42], [43], [44]. Where spatial variation in isotopic composition of prey can be dismissed, comparisons of trophic niche widths may be possible e.g. as in Willson et al. [40] who used a small, isolated study site to investigate aquatic snakes. Unfortunately for the majority of habitats and study species, it is clear that a detailed knowledge of species-specific feeding behaviour and the ecology of the community are required before variability in prey isotopic composition can be identified and accounted for [1], [15], [45]. The use of multiple methods may aid the accuracy of estimation of trophic niche width using stable isotopes, and as such, a number of studies have successfully utilised the information from stable isotopes combined with gut analysis to make informed estimates of trophic niches [42], [46], [47].

The ‘niche variation hypothesis’ proposed by Van Valen [11] predicts that “populations with wider niches are more variable than populations with narrower niches” [48]. Correspondingly, Bearhop et al. [12] predicted that populations consuming a wider range of prey and those that forage in a range of geographical areas could display wider isotopic variation than those that have a narrow range of prey and limited foraging capacity. In accordance with Bearhop et al's [12] predictions, Olsson et al. [41] examined the isotopic niche widths of invasive and native crayfish in Swedish streams. The greater niche width of the introduced species reflected a wider use of habitat and/or prey sources. However at the population level, the two species did not differ in niche widths, indicating that isotopic variability between habitats was confounding any differences [41]. Accordingly, our models have identified the confounding influence of habitat use on predictions of trophic niche width. Furthermore, comparisons of populations of dietary generalists feeding on the common four prey sources indicate that isotopic variation among habitat specialists was similar or greater than the equivalent habitat generalists (Table 3). Niche width may increase by either the entire population shifting to use all available resources or by an increase in inter-individual specialisation within a population (see [49]). Simulations of populations of dietary generalists here suggest that populations confined to one site may display greater isotopic variance within their population due to individual specialisation. This individual niche variation among conspecific individuals has been suggested as being widespread [49], indicating that the variation in isotopic niche within a population may further confound any estimates of a populations trophic niche width. For example, predators within the same population with different dietary specialisations can account for greater trophic variability at the population level than the same population composed of generalists.

Fundamentally, anything which prevents or causes an organism to sample only a portion of the complete distribution of prey signatures where variation exists could result in incomplete and inaccurate estimates of niche width. Our data indicates that as the variance in prey items increases, the greater there is for the potential of inaccuracy (dependant on the spatial distribution of the signatures). The influence of distance between resources on the foraging behaviour of animals has been well established [50], [51], and such effects may be driven by macronutrient regulation [52], [53] and prey availability [54]. Data from simulated populations of Almaco Jack accounting for distance between foraging locations revealed that isotopic values were variable and prey species-dependent. Many communities are vastly more complex than a four prey model [49] and large predators are likely to feed on a greater diversity of prey [55]. Inclusion of all prey species of a similar trophic level to the model, to further increase ecological realism, showed that habitat generalists displayed narrower niches than habitat specialists. Dietary specialists will typically exhibit a broader trophic niche than dietary generalists because they lack the influence of different prey items that are variable in their isotopic signature. That is across many sites where variation in prey signatures exists, the range between means will be less for predators that eat multiple prey items (dietary generalist) than for those that only eat specific prey (dietary specialists).

This problematic nature of estimates of niche width using variance in δ-space has been addressed by Newsome et al. [19], who proposed the use of mixing models to transform data into p-space (dietary proportions). The transformation provides a value comparable to other common variables used in studies of ecological niches, and corrects for magnitude differences amongst isotopic composition of prey [19]. In the present study the mixing models reduced the variances observed in p-space (Table 6) compared with those observed in δ-space (Table 3), however, they failed to reduce the differences in variances observed amongst models of the Alcamo Jack populations. In both parts of the study (1 & 2) where variances were compared in both δ-space and p-space, it was clear that this transformation maintained and in many instances increased the observed differences in isotopic variances between the simulated models (Table 7). We therefore concur with the findings of Flaherty and Ben-David [15] who raised concerns with the use of such transformation. Furthermore, many mixing models used to estimate proportional values are reliant on amounts of a priori information, in such cases isotopic mixing models are sometimes less informative than non-isotopic information in its raw form i.e. stomach content data (see [1] for discussion).

Flaherty and Ben-David [15] modelled the attempts of a “naive researcher” who ignores habitat use of the study species when using isotopic data to estimate the trophic niche. In a similar manner, we used mixing spaces [32] to reproduce these simulations within a marine ecosystem. In comparison, mixing spaces for habitat specialists (D_g_H_s_ and D_s_H_s_) were defined using source values from each site. If habitat variability in isotopic signatures is an important source of variation [15], [16], [56], it seems only appropriate that we define mixing space accordingly. Like Flaherty and Ben-David [15], we too encountered many isotopic values that fell outside of the mixing space. Because simulations are based on the isotopic signatures of the global or site mean of the prey species, when populations of specialist predators are observed a large proportion of the calculated isotopic values will fall outside their mixing space, independent of mixing space width. As variability in δ^13^C and δ^15^N of the primary producers in food webs exist among habitats [57], [58], [59], comparisons of δ^13^C and δ^15^N among habitats will be confounded by isotopic variability of the prey source [3].

Mixing models that provide estimates of prey item proportions within diets are becoming popular to determine partitioning of dietary resources. Such models have been refined [32], [34], [60], [61] and debated [62], [63] over recent years. Very recent examples of their use include Kristensen et al. [64], who applied mixing models to δ^13^C and δ^15^N isotopes to determine resource partitioning amongst leaf-eating mangrove crabs, and Flaherty et al. [65] used similar models to determine the contribution of different prey items to overall diet of flying squirrels. We tested and compared numerous models to determine if the partitioning of a resource by populations could be identified. It can be seen that in the majority of cases SISUS and IsoSource made very similar estimates, but different to those from the MixSIR and SIAR models (Table 6). The mixing models all predicted that Almaco Jack fed in a relatively generalist manner on all four prey species, with the exception of the SIAR model for D_s_H_s_ in Part 1. This includes models generated in part 2 for dietary specialists (D_s_H_g_ and D_s_H_s_), which were simulated to feed exclusively on *P. scabriusculus*. Of concern was that on closer inspection of the proportions estimated, it was evident that no mixing model was able to accurately estimate proportions of the dietary specialists, possibly with the exception of SIAR for D_s_H_s_, irrespective of isotopic variation of habitats (Table 6). For part 2, SIAR failed to allocate the majority of the diet to the specialist prey item, *P. rubrizonatus*.

Transformation of the data to dietary proportions failed to distinguish the correct partitioning of prey sources for dietary specialists. In Part 1 mean estimates among mixing models for predators specialising on *P. scabriusculus* determined that this prey source, only counted for approximately a ¼ of their diet irrespective of the habitat model. In part 2, mean estimates amongst mixing models for predators specialising on *P. rubrizonatus* revealed that *P. rubrizonatus* accounted for only 31% of their diet, while other “uneaten” individual prey species contributing up to 49% of the diet (Table 6). Because no mixing model was able to accurately estimate proportions of the dietary specialists, irrespective of isotopic variation of habitats (Table 5), our data therefore show that inaccuracies amongst estimates provided by linear mixing models may go well beyond problems associated with habitat variability.

Like Flaherty and Ben-David [15], we too provide simplistic approaches to what are in reality, much more complex systems [49] that are likely to substantially underestimate the true extent of isotopic variability. We have attempted to include greater ecological complexity by including foraging distance and by using entire assemblages across a trophic level as prey sources. With these additions our models show that isotopic variability amongst habitats will confound estimations of trophic niche derived from measures of isotopic niche width in both δ-space [12] and p-space [19]. While the variability of prey isotopes is lower than may be encountered in some ecological systems but still likely reflective of many, it remains clear that isotopic niche is not a reliable indicator of trophic niche. Of greater concern was the failure of mixing models to correctly identify dietary specialisations and potential resource partitioning. Additionally, our simulations bring into question the accuracy of mixing models in identifying contribution sources, irrespective of whether isotopic variability amongst habitats exists. Our findings emphasise that progress in isotopic studies in animal ecology will require a greater understanding of the functional traits and behaviour of organisms.

## Supporting Information

Figure S1 Data output from simulations of the isotopic signatures for Part 1 from the modelled Almaco Jack in δ-space that were both dietary and habitat specialists (D_s_H_s_) for the common.
(TIF)

Figure S2 Data output from simulations of the isotopic signatures for Part 1 from the modelled Almaco Jack in δ-space that were both dietary and habitat specialists (D_s_H_s_) for the common species.
(TIF)

Figure S3 Data output from simulations of the isotopic signatures from the modelled Almaco Jack in δ-space. A) Habitat generalists specialising on the common species (D_s_H_g_) accounting for distance between sites – Part 2. B) – Habitat specialists feeding on the entire prey assemblages (D_g_H_s_) – Part 3.
(TIF)

Text S1 Detailed model description.
(DOCX)
