# Supplementary figures and images for: Estimating Niche Width Using Stable Isotopes in the Face of Habitat Variability: A Modelling Case Study in the Marine Environment
Source: PLoS One. 2012 Aug 2;7(8):e40539. doi: 10.1371/journal.pone.0040539 (PMC3410910; doi:10.1371/journal.pone.0040539)

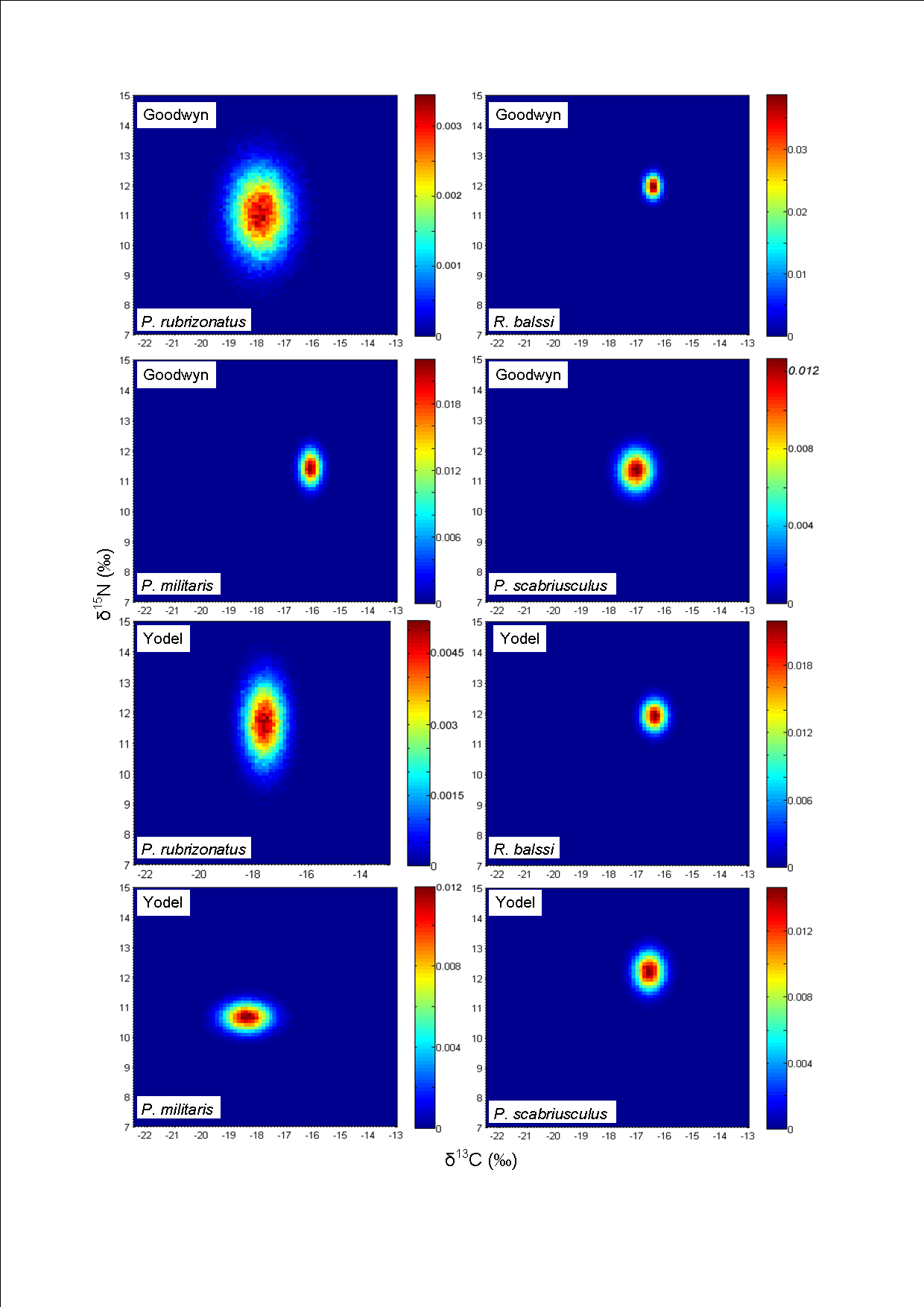

Supplement: Figure S1 — Data output from simulations of the isotopic signatures for Part 1 from the modelled Almaco Jack in δ-space that were both dietary and habitat specialists (DsHs) for the common. (TIF) [file pone.0040539.s001.tif]

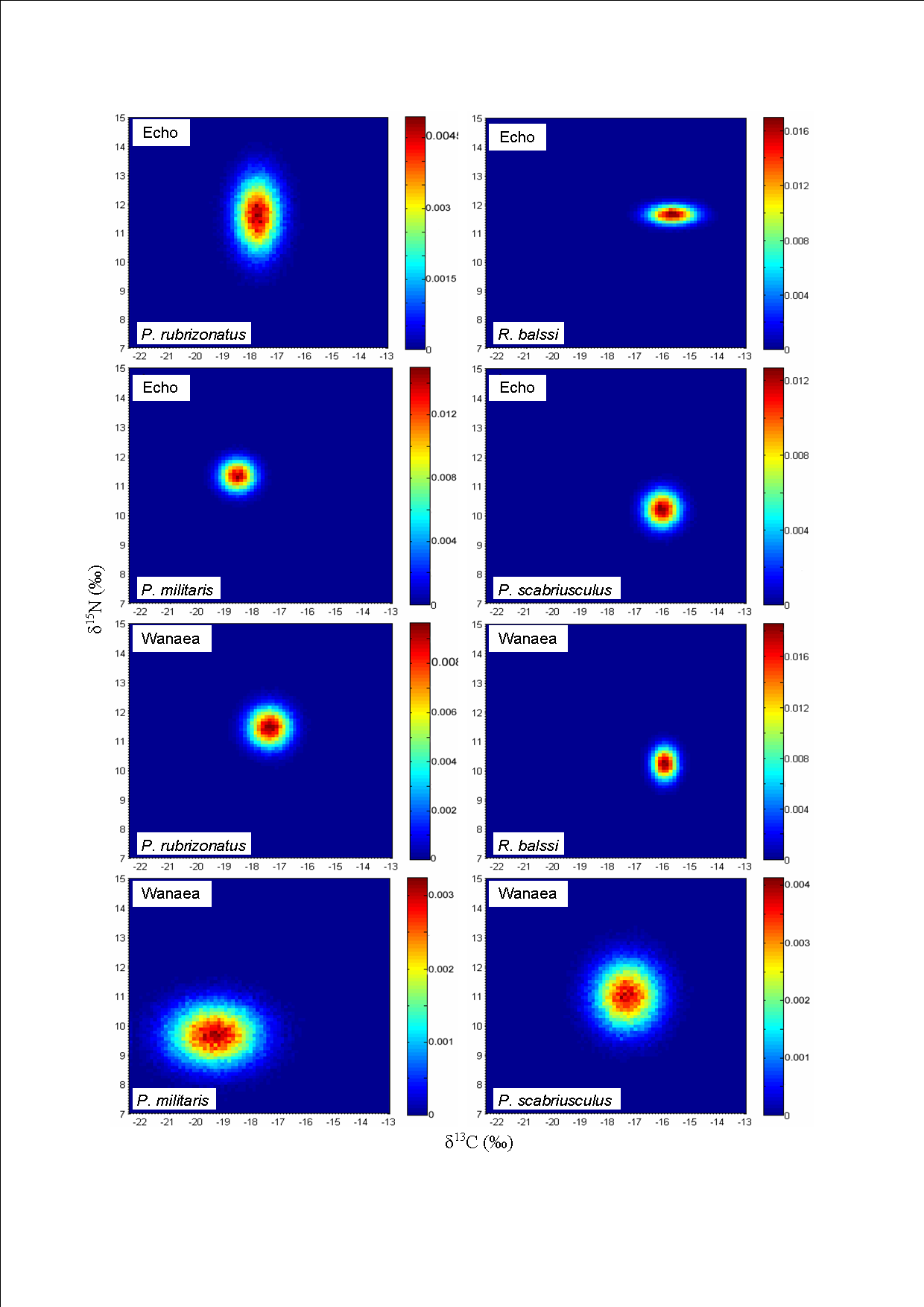

Supplement: Figure S2 — Data output from simulations of the isotopic signatures for Part 1 from the modelled Almaco Jack in δ-space that were both dietary and habitat specialists (DsHs) for the common species. (TIF) [file pone.0040539.s002.tif]

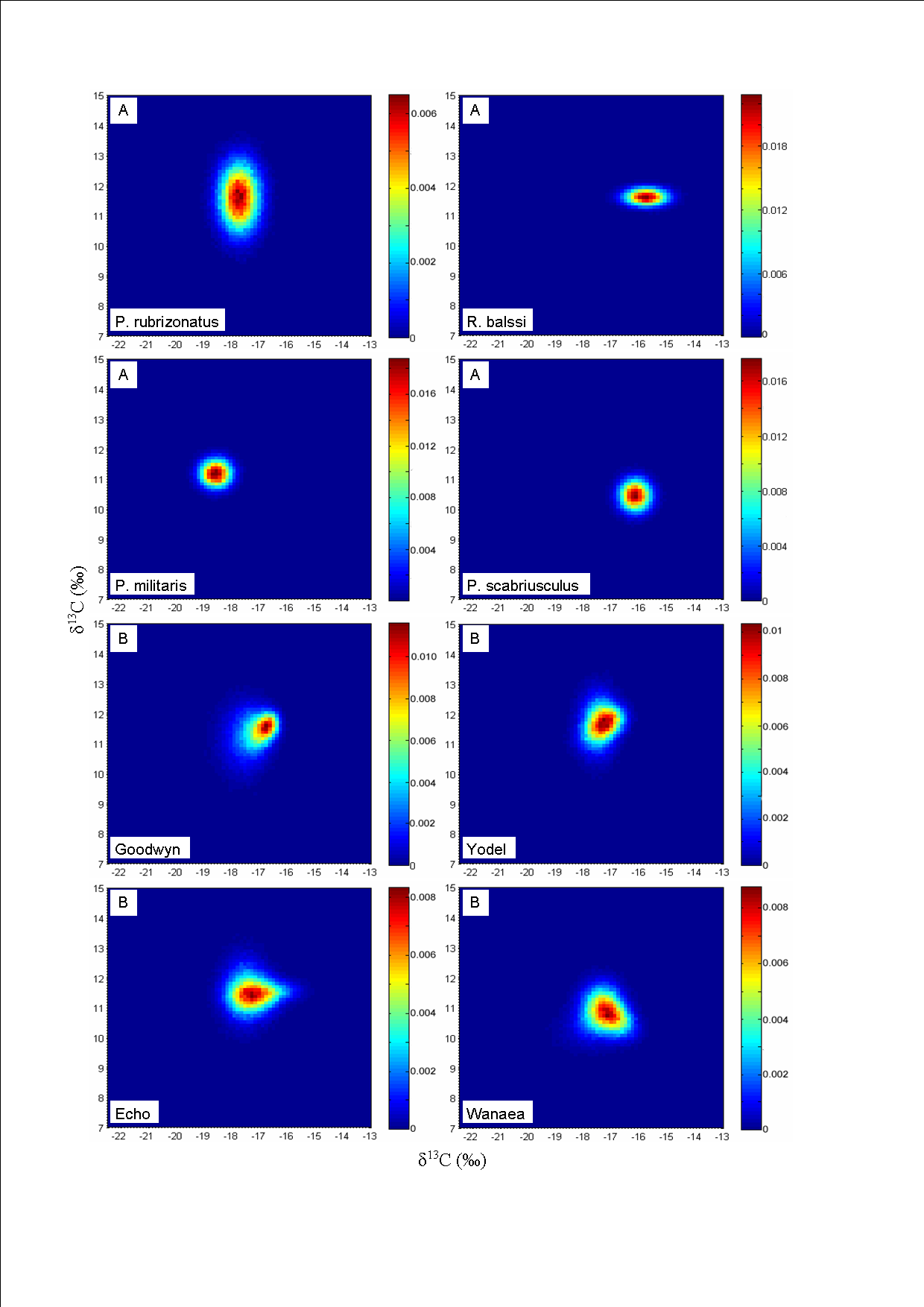

Supplement: Figure S3 — Data output from simulations of the isotopic signatures from the modelled Almaco Jack in δ-space. A) Habitat generalists specialising on the common species (DsHg) accounting for distance between sites – Part 2. B) – Habitat specialists feeding on the entire prey assemblages (DgHs) – Part 3. (TIF) [file pone.0040539.s003.tif]
