# Supplementary material for: Estimating Niche Width Using Stable Isotopes in the Face of Habitat Variability: A Modelling Case Study in the Marine Environment
Source: PLoS One. 2012 Aug 2;7(8):e40539. doi: 10.1371/journal.pone.0040539 (PMC3410910; doi:10.1371/journal.pone.0040539)
Supplement: Text S1 — Detailed model description. (DOCX) [file pone.0040539.s004.docx]

**Text S1:**

The model used here was inspired by the one formulated by Flaherty and Ben David [15]. In this model, a forager consumes one (diet specialist Ds) or a combination (diet generalist Dg) of species in one (habitat specialist Hs) or a combination (habitat generalist Hg) of sites, and we consider all combinations of the above alternatives with 100 000 replicates per condition (e.g. there are 4 sets of 100 000 replicates for a D_g_H_s_ scenario, with one set per habitat). Using our empirical data, we model 4 habitats corresponding to the 4 study sites. Part 1 and 2 of the model consider 4 species that are present in all the habitats, with differences residing in how we define the relative contribution of habitats for generalists. For Part 3, we focus on diet generalists where each habitat has its own set of prey species.

When the forager consumes a species *j* in the habitat *i*, the prey contributes to the forager ^13^C and ^15^N isotope signatures by the amounts $\delta^{13}C_{i}^{j}$ and $\delta^{15}N_{i}^{j}$ respectively, which are determined by randomly generating a number from a Gaussian distribution of mean $\mu$ and standard deviation $\sigma$ taken from the empirical data for the corresponding species and site. For a habitat and dietary specialist foraging in habitat *i* and consuming the species *j*, the final forager isotope signature will be equal to $\delta^{13}C_{i}^{j}$ and $\delta^{15}N_{i}^{j}$, while a forager which is generalist in either or both habitat and species, relative contributions for species and/or habitats are determined by using the rules detailed below.

In part 1 and 3, as well as for species within an habitat in part 2, the contribution of each of the *M* habitats available or *S_i_* available species (within habitat *i*) is a random fraction. For example, a species generalist in a given habitat *i* will randomly forage on *S* available species with each of species *j* contributing by a random fraction $\theta_{j}$, with $\sum_{j=1}^{S_{i}} \theta_{j}=1$.

The resulting signatures inherited at the habitat level at site *i* will be

$\delta^{13}C_{i}=\sum_{j=1}^{S_{i}} {\theta_{j}\times\delta}^{13}C_{i}^{j}$ and $\delta^{15}N_{j}=\sum_{j=1}^{S_{i}} {\theta_{j}\times\delta}^{15}N_{i}^{j}$ (1)

If the forager is also a habitat generalist, then each habitat *i* out of the *M* habitats will also contribute by a random fraction $\rho_{i}$, with $\sum_{i=1}^{M} \rho_{i}=1$.

The resulting signature for the individual will be

$\delta^{13}C=\sum_{i=1}^{M} {\rho_{i}\times\delta}^{13}C_{i}$ and $\delta^{15}N=\sum_{i=1}^{M} {\rho_{i}\times\delta}^{15}N_{i}$ (2)

In part 2, the rules that determine the relative contribution of each habitat are modified to take into account distances between patches. To achieve this, we simulate the movement of a forager between each patch using the following set of rules. Each individual forages for 500 time steps. The habitat in which it starts is chosen randomly. Then, at each new step, the individual chooses to move from habitat *j* to another habitat *i* with the probability *P_ij_* for this habitat to be chosen,

$P_{ij}=\frac{d_{ij}^{-1}}{\sum_{k=j,k\neq j}^{M} d_{kj}^{-1}}$ (3)

where $d_{ij}$ represents the distance between the habitat *j* where the predator currently is and the habitat *i*. At the end of the 500 steps, the fraction $\rho_{i}$ allocated to the habitat *i* corresponds to $\rho_{i}={t_{i}}/{500}$, where *t_i_* is the number of times the predator chose to forage in the habitat *i*. These fractions are then used to calculate the individual signatures as stated in equation (2).
